# Prediction of PCOS and Mental Health Using Fuzzy Inference and SVM

**DOI:** 10.3389/fpubh.2021.789569

**Published:** 2021-11-30

**Authors:** Ashwini Kodipalli, Susheela Devi

**Affiliations:** ^1^Department of Computer Science and Automation, Indian Institute of Science, Bengaluru, India; ^2^Department of Artificial Intelligence and Data Science, Global Academy of Technology, Bengaluru, India

**Keywords:** support vector machines, fuzzy TOPSIS, fuzzy AHP, polycystic ovarian syndrome, mental health issues, machine learning, classifiers, fuzzy logic

## Abstract

Polycystic ovarian syndrome (PCOS) is a hormonal disorder found in women of reproductive age. There are different methods used for the detection of PCOS, but these methods limitedly support the integration of PCOS and mental health issues. To address these issues, in this paper we present an automated early detection and prediction model which can accurately estimate the likelihood of having PCOS and associated mental health issues. In real-life applications, we often see that people are prompted to answer in linguistic terminologies to express their well-being in response to questions asked by the clinician. To model the inherent linguistic nature of the mapping between symptoms and diagnosis of PCOS a fuzzy approach is used. Therefore, in the present study, the Fuzzy Technique for Order of Preference by Similarity to Ideal Solution (TOPSIS) method is evaluated for its performance. Using the local yet specific dataset collected on a spectrum of women, the Fuzzy TOPSIS is compared with the widely used support vector machines (SVM) algorithm. Both the methods are evaluated on the same dataset. An accuracy of 98.20% using the Fuzzy TOPSIS method and 94.01% using SVM was obtained. Along with the improvement in the performance and methodological contribution, the early detection and treatment of PCOS and mental health issues can together aid in taking preventive measures in advance. The psychological well-being of the women was also objectively evaluated and can be brought into the PCOS treatment protocol.

## Introduction

Polycystic ovarian syndrome (PCOS) is a hormonal disorder usually found in young adult women and in women of reproductive age. It is a common and pervasive hormonal disorder with multiple phenotypes having different presentations (1). PCOS was first discovered by Stein and Leventhal (2). According to WHO, around 6–26% of women are affected by PCOS. It includes failure to ovulate and infertility, along with mental health complications. PCOS has several effects ranging from acne and obesity to irregular menstruation and infertility, which might lead to imparity in the quality of life (3–5). PCOS can be detected using the Rotterdam Consensus, where the person under study should meet at least two of these conditions: (a) absence of ovulation, (b) symptoms of hyperandrogenism, and (c) ovaries affected with polycysts (6). PCOS causes several effects such as amenorrhea, obesity, type 2 diabetes, metabolic impairment, and cardiovascular disorders. As per WHO, the prevalence of PCOS is estimated to be between 6 and 26% (7). In India, the prevalence of PCOS ranges between 9.13 and 36% (8). PCOS is one of the major factors for infertility and emotional problems. Approximately up to one-third of females with PCOS have fertility issues. Women with PCOS have more anxiety and depression than women in general. The percentage of depression and anxiety in women with PCOS is 28–39% and 11–25%, respectively (9, 10). International research shows that PCOS has a significant effect on both physical and emotional well-being and, hence, affects the quality of life (11).

Recent research has shown that PCOS can be detected using an ultrasound scan where the doctor counts the size and number of follicles in the ovaries. PCOS can also be detected using biochemical examinations such as blood tests and hormonal examinations, yet it is an expensive investigation. It can also be predicted using clinical parameters such as menstrual cycle length and body mass index (BMI), however, it involves clinical acumen and the subjectivity of the clinician, as well as the intrusion of privacy of the women; also, the process is not automated and the emotional wellness of the women is not considered.

In the literature, various methods are proposed to detect PCOS. They are broadly categorized into two groups.

1. The studies where the association between PCOS and mental health is investigated using various statistical tools such as χ^2^ and Fisher's exact test. Here, however, the diagnosis of PCOS and its accuracy are not considered.
2. The studies where PCOS is detected using machine learning algorithms and image processing techniques, but psychological and emotional wellness is not considered.

We were motivated by the above limitations of existing works; thus, the current research is designed to include both clinical and psychological parameters to detect and diagnose the likelihood of having PCOS and its associated mental health problems. Since the clinical and psychological data pertaining to PCOS varies from woman to woman and involves inherent uncertainty, imprecision, and ambiguity, the traditional machine learning algorithms will not model the system under consideration. The fuzzy algorithms such as the fuzzy technique for order of preference by similarity to ideal solution (TOPSIS) and fuzzy analytical hierarchal process (AHP) are evaluated for their efficacy and robustness against traditional algorithms such as support vector machines (SVM), K nearest neighbor, and decision tree (D-tree).

The organization of the paper is as follows. Related work describes the related work, the drawbacks of the existing methods, and the problem statement. Proposed method provides the proposed methodology and collection of data. Results explains the results and Discussion includes the comparative study of the results through discussion. Finally, Conclusion concludes the paper.

## Related Work

Some of the studies which proved the relationship between PCOS and mental health and the classification algorithms used for the prediction of PCOS are reviewed and presented below:

As per the systematic and exhaustive survey conducted by Himelein et al. (12), it was indicated that PCOS is also associated with psychological/mental health issues such as body dissatisfaction, depression, anxiety, and eating disorders, and thus, reduces the quality of life. Although in his research he did not describe the implicit relationship between PCOS and mental health, he suggested strongly that the effective treatment of PCOS can stabilize mental health issues.

Conte et al. (13) conducted a systematic review on mental health and physical exercise in women with PCOS and reported that introducing physical activity can improve the quality of life and psychological well-being of women. Kerchner et al. (14) established the relationship between PCOS and mental health and identified the risks and predictors for depression in women with PCOS. In his research, a total of 60 women with PCOS were considered and were given Primary Care Evaluation of Mental Disorders Patient Health questionnaires (PRIME-MD PHQ) (15), the Beck Depression Inventory-II (BDI) (16), and the Beck Anxiety Inventory (BAI) (17) to detect mental health disorders. The data were analyzed using χ^2^ and Fisher's exact test to evaluate the categorical variables. It was found that 40% (24 out of 60) of women are suffering from depression and 16.6% suffer from mood disorders which shows that at least 56.6% of women with PCOS have mental health issues.

Wolf et al. (18) conducted a systematic review based on geographical location and ethnicity. The study found that the prevalence of PCOS was between 6 and 9% across the United States, United Kingdom, Spain, Greece, Australia, Asia, and Mexico based on the National Institutes of Health (NIH) diagnostic criteria. The study also suggested that there is no racial or ethnic influence on the prevalence of PCOS. Naz et al. (19), demonstrated the prevalence of PCOS in the adult population. In his meta-analysis, 12 published articles were considered and Egger and Begg's tests were used to check the publication bias. Using 477 participants and the STATA software, it is proved that PCOS in the adult population was 11.04% based on the Rotterdam criteria, 3.39% based on the NIH criteria, and 8.03% based on the Androgen excess and PCOS society.

Banting et al. (20), in their research along with the association of mental health and PCOS, also investigated physical inactivity, motivators, and support providers for women with and without PCOS. Using a local database and computational statistics, he concluded that a woman with PCOS requires more support compared with women without PCOS. Similarly, Berni et al. (21), investigated Autism Spectrum Disorder (ASD) and Attention Deficit Hyperactivity Disorder (ADHD) and proved that the incidence of ASD and ADHD is higher in children of mothers with PCOS. In a similar development, Chandhari et al. (22) investigated the association of PCOS with psychiatric morbidity and its impact on quality of life. Using binary logistic regression, he could associate PCOS with psychiatric morbidity.

Some studies investigated the detection of PCOS using computational and machine learning algorithms. In a recent development, Sachdeva et al. (23) compared the phenotypes in PCOS by considering their metabolic, clinical, and hormonal profiles, and their differential responses to clomiphene. Using descriptive statistics, the authors concluded that patients with full-blown PCOS (phenotype A) are at a higher risk of metabolic and cardiovascular disorders compared with others, thus concluding that phenotype division can help patients in their accurate diagnoses. In a recent computational study by Denny et al. (24), an early detection and prediction model for PCOS was proposed and it was concluded that the random forest classifier provided an accuracy of 89.02%. He considered 541 women with 23 attributes that consist of both clinical and metabolic parameters. Similarly, another work by Vikas et al. (25) considered 119 women with 18 attributes consisting of clinical parameters that were used to evaluate PCOS and obtained an accuracy of 97.65% using the Naïve Bayes classifier. The study conducted by Anuradha and Priyanka (26) considered 84 women and 13 attributes to detect PCOS and obtained an accuracy of 94% using artificial neural networks. The study by Deshpande and Wakankar (27) considered imagining parameters such as follicles along with the biochemical and clinical parameters such as hormonal levels and BMI for the detection of PCOS. Support vector machine was used for the classification and obtained an accuracy of 95%.

Mishra and Prakash (28) found that, for computing human cognitive capabilities, fuzzy logic is the most effective computing method. The categories of the medical data are clearly explained in this paper. Further, Ansari et al. (29), explained in their research how Fuzzy TOPSIS can be used extensively for trustworthy health care development software.

But none of the studies have considered mental health in their analysis. In all the studies, the data was obtained by interviewing the women and from the laboratory. As the characteristics of the data are uncertain and since the likelihood of having PCOS depends on multiple parameters, fuzzy algorithms are more appropriate than crisp algorithms.

The following lacunae were found from the literature survey:

1. The studies where the association between PCOS and mental health is investigated did not evaluate the performance, efficacy, and robustness of PCOS detection.
2. Among the studies where the PCOS detection is carried out using machine learning algorithms, the psychological well-being parameters are not considered.
3. Integrated studies on PCOS detection and the analysis of its associated mental health were not present in the literature.
4. The existing studies employ statistical tools. However, there are no studies that could implement soft computing approaches like fuzzy sets and multi-criteria decision analysis systems.
5. An effective and early diagnosis system that can screen many people in a time-bound and cost-effective manner does not exist.
6. There are limited computer-assisted tools that help women in the screening of PCOS and its associated mental health privately and comfortably. It only requires the woman who is to be tested to take the questionnaire and get the diagnosis immediately.

In the current study, both physical and psychological parameters are considered for developing an automated PCOS and its associated mental health detection. The clinical data collected from the diverse population is non-deterministic, irregular, imprecise, and uncertain, making soft computing approaches such as fuzzy TOPSIS and fuzzy AHP better candidate methods than traditional methods.

## Proposed Method

The methodology proposed in this study is described below:

### Study Design

The research is mainly focused on developing an early prediction model. The study is designed to include young adult women of age <25. Women taking treatment for acute illness, pregnant women, and women under substance abuse are excluded from this study. The study protocol was approved by the ethical committee of the institution where the study is carried out. The written consent of the participants (subjects) was collected before involving them in the study. The study involved the participation of two expert gynecologists and one psychologist who designed the questionnaires. The questionnaire included 11 questions related to physical aspects, 10 questions related to anxiety and depression using the K10 tool, 5 questions on social phobia, and 5 questions on body image dissatisfaction. Table 1 provides a brief overview of this questionnaire. The expert panels comprising of two gynecologists and one psychologist interviewed the subjects and filled the questionnaire form. Based on the answers for different questions, the expert panels have mapped the subjects into one of the four categories, viz, A1: subject having both PCOS and mental health issues; A2: subject having only PCOS problem; A3: subject having only mental health issues; and A4: subject being normal. The expert panel filled the questions by interviewing the subjects and also mapped every subject to one of the four predefined categories.

**Table 1:** Brief overview of the questionnaire.

**Variables**	**Criteria**
**Physical aspects**	
C11	Regularity of periods
C12	Length of the menstrual cycle
C13	Duration of the flow
C14	Number of pads used per day
C15	During cycle, tendency to grow dark, coarse hair on chest and chin
C16	Weight gain
C17	Eating junk food
C18	Meal times and eating pattern
C19	Sleep Schedule/Sleep pattern
C110	Family history of diabetes
C111	Family history of hypertension
**Anxiety and depression (K10 Tool)**	
C21	Feel tired for no good reason
C22	Feel nervous
C23	Feel so nervous that nothing could calm you down
C24	Feel hopeless
C25	Feel restless or fidgety
C26	Feel so restless that you could not sit still
C27	Feel depressed
C28	Feel everything was an effort
C29	Feel so sad that nothing could cheer you up
C210	Feel worthless
**Social phobia**	
C31	Intense and persistent fear that others might evaluate you
C32	Fear of being humiliated in social situations
C33	Feeling extremely self-conscious
C34	Fear that others will notice blushing/sweating
C35	Try hard to avoid social situation/interaction
**Body image dissatisfaction**	
C41	Spend a lot of time worrying about their appearance
C42	Experience dissatisfaction with their appearance
C43	Avoid wearing certain cloths because they may look fat
C44	Compares their appearance with others and feel low
C45	Dissatisfaction and self-consciousness of appearance interferes with social activities and interactions

### Data Collection

**Figure 1:**
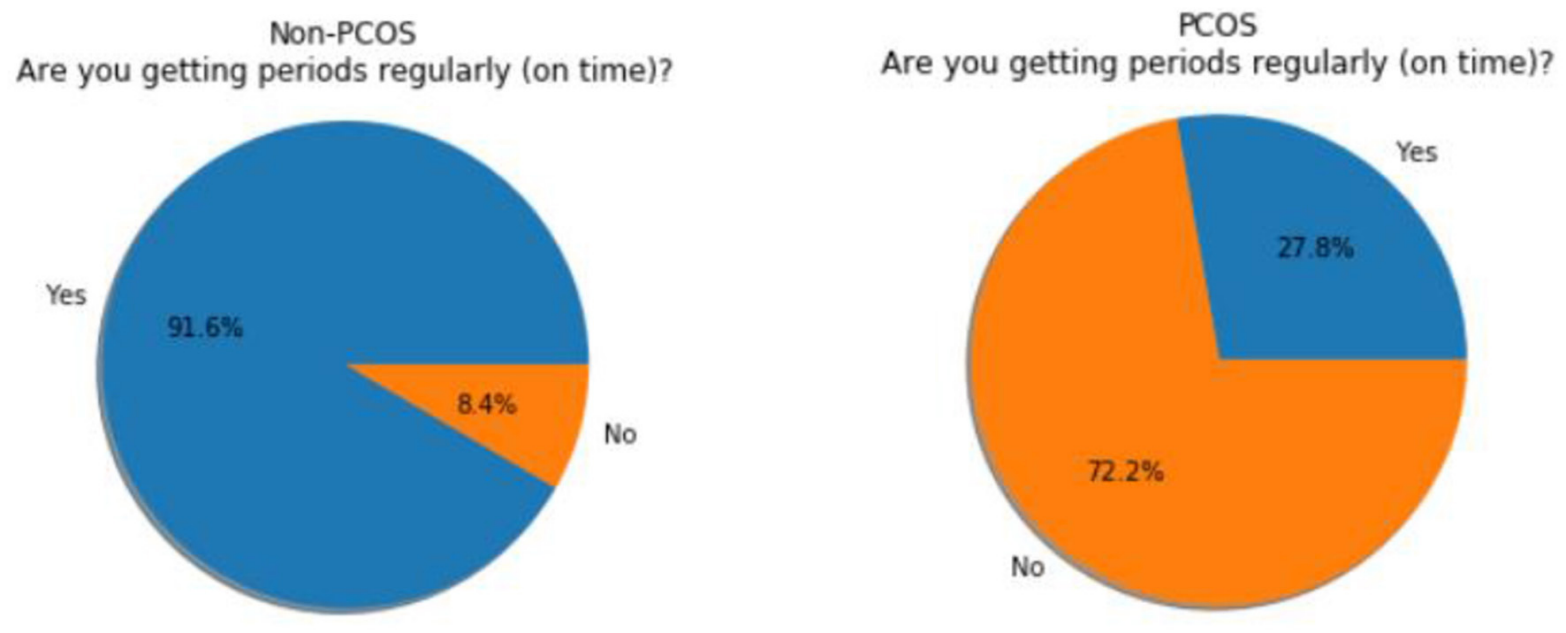
Non- polycystic ovarian syndrome (PCOS) subjects vs. PCOS subjects (regularity in the cycle).

**Figure 2:**
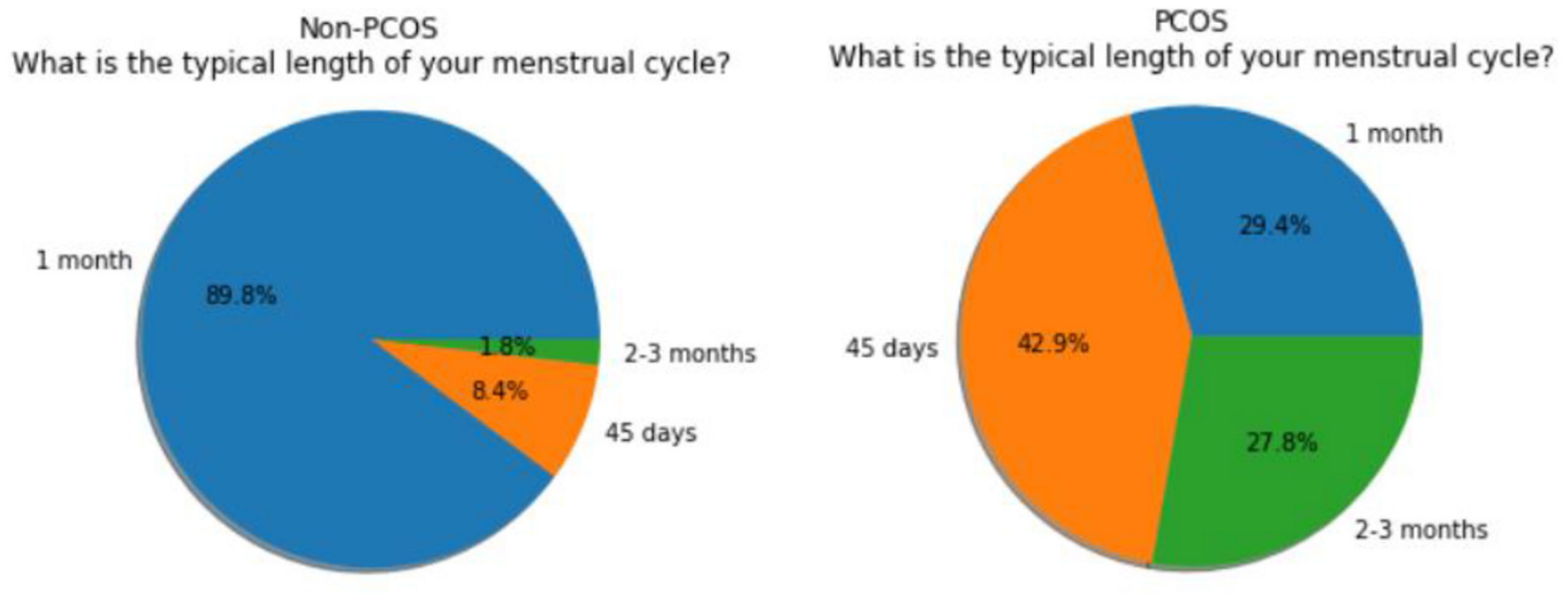
Non-PCOS subjects vs. PCOS subjects (typical length of menstrual cycle).

In total, 660 subjects were chosen for the study where 624 have agreed to participate and others have declined to participate due to various reasons. The expert panel completed the questions by interviewing the subjects and also mapped every subject to one of the four pre-defined categories. This data is stored in a comma-separated values (CSV) file and it is used and processed for subsequent analysis by algorithms. Data visualization and data analysis, using matplotlib, were performed to gain more insight into the data. After analysis, the results of the regularity in the menstrual cycles of the subjects are shown in the form of a graph in Figure 1. The pie chart of the answers of the subjects regarding the length of their menstrual cycle is shown in Figure 2.

### Methodology

**Figure 3:**
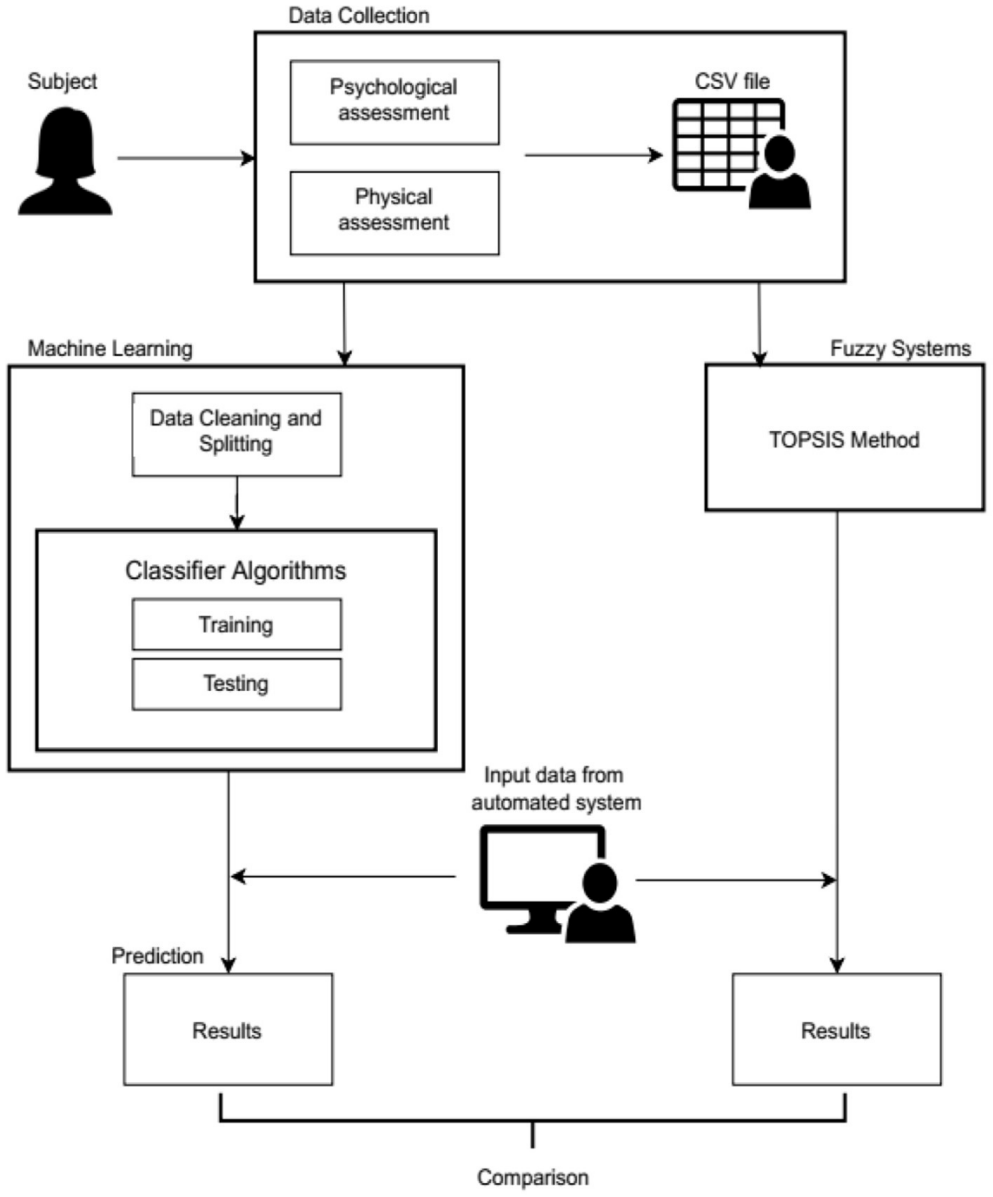
Block diagram of the proposed method.

As noted in the literature survey in the previous section, the majority of research in this area is carried out using traditional methods like the T-square test, Chi-Square test, etc. The current implementation is an approach to apply machine learning and fuzzy systems to the data and perform a comparative study of the two. Figure 3 depicts the block diagram of the proposed method.

A woman who has agreed to participate in the study is called a subject under study. The data collection for this study includes both physical assessments (menstrual cycle, regularity of the cycle, length of the cycle, duration of the cycle, recent weight gain, hair loss, family history of having diabetes and hypertension, and eating and sleeping habits) and psychological assessments (anxiety, depression, body image dissatisfaction, and social phobia). Based upon the answers given by the subjects to the 31 questions, the panel of doctors decides to put them into one of the four categories. Once the data is collected, it is stored in a CSV file. The data pertaining to each subject corresponds to a row and their answers for each question are noted in the corresponding columns. Thus, the dataset has 31 columns i.e., one column for every question and one last column for the category they fall into. This file is then used to implement classifier algorithms in the machine learning and TOPSIS method in the Fuzzy systems.

In the machine learning module, the acquired data in the CSV file undergoes data processing, which includes checking for missing and erroneous values. As the responses chosen by the subject are categorical, the values are replaced with integers for the representation and processing by machine learning algorithms. Eighty percent of the data is used for training and the remaining 20% of the data is used as test data. After analyzing the training data, it is observed that the data is not balanced as few classes had fewer numbers of samples and the remaining classes had more numbers of samples. Therefore, the data is balanced using the K nearest neighbor algorithm. Some samples are chosen and their K nearest neighbors are found and the mean values of these samples are added to the training data. The first 31 columns are considered as the features and the last column is the target variable. A classifier is used to predict the category of the new instances. The algorithms used here are the D-Tree classifier, K-Nearest Neighbor (KNN) classifier, and the SVM as they are some of the most commonly used classifiers in the domain. The algorithms use the training data to learn. The prediction is performed on the test data. The models are evaluated by checking their accuracy scores. An automated system is also a part of the implementation which is used to take a new instance as input by entering options in the set of questions. This is fed to the classifier to predict the appropriate class of the new instance.

Similarly, the same CSV file is used to implement the TOPSIS method. For the implementation using the fuzzy TOPSIS method, the data in the original form is considered without converting the response (for example, less than a day, more than 7 days, 5–7 days, quite irregular, fairly irregular, sometimes, most of the times, etc.) into numerical form, where the response given by the subjects are uncertain or vague.

**Figure 4:**
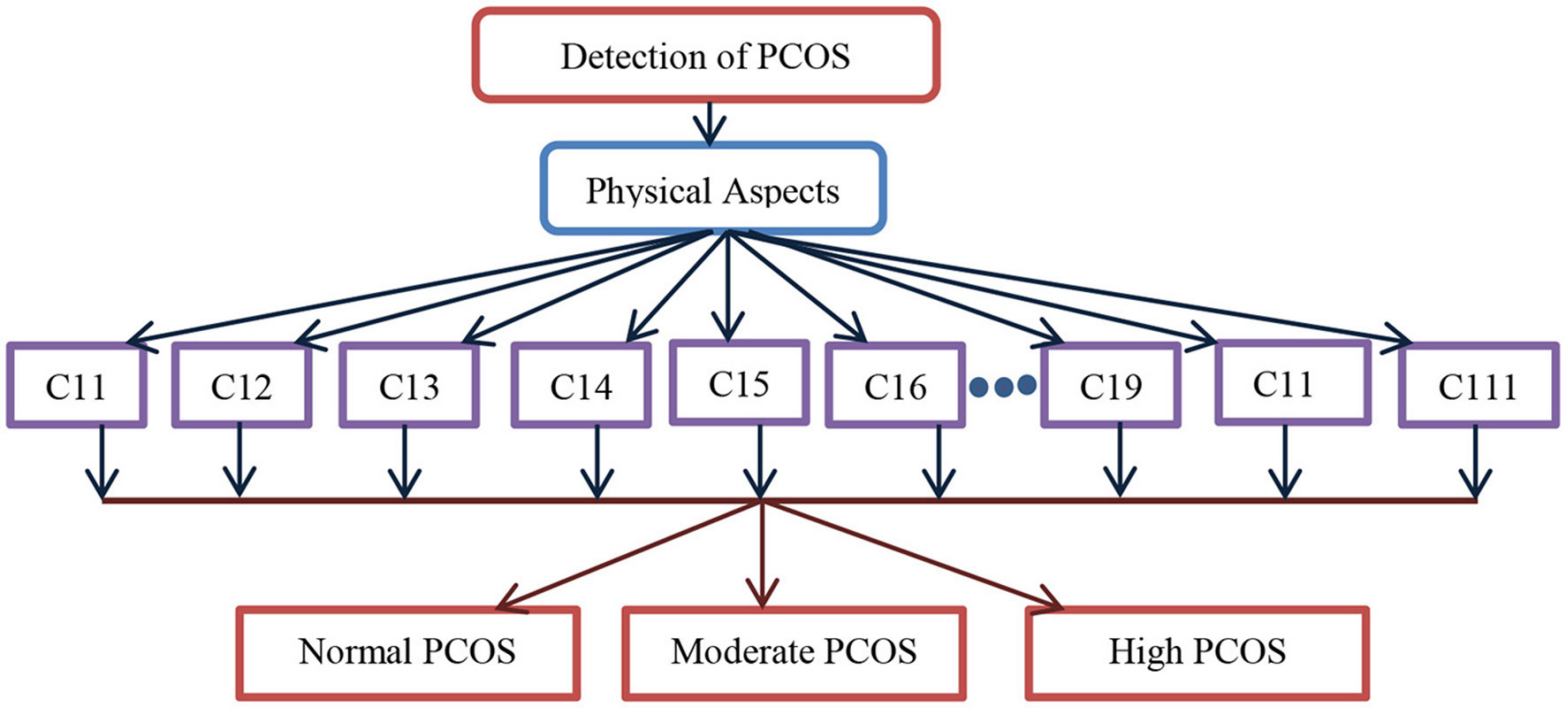
The computational model for the detection of PCOS.

**Figure 5:**
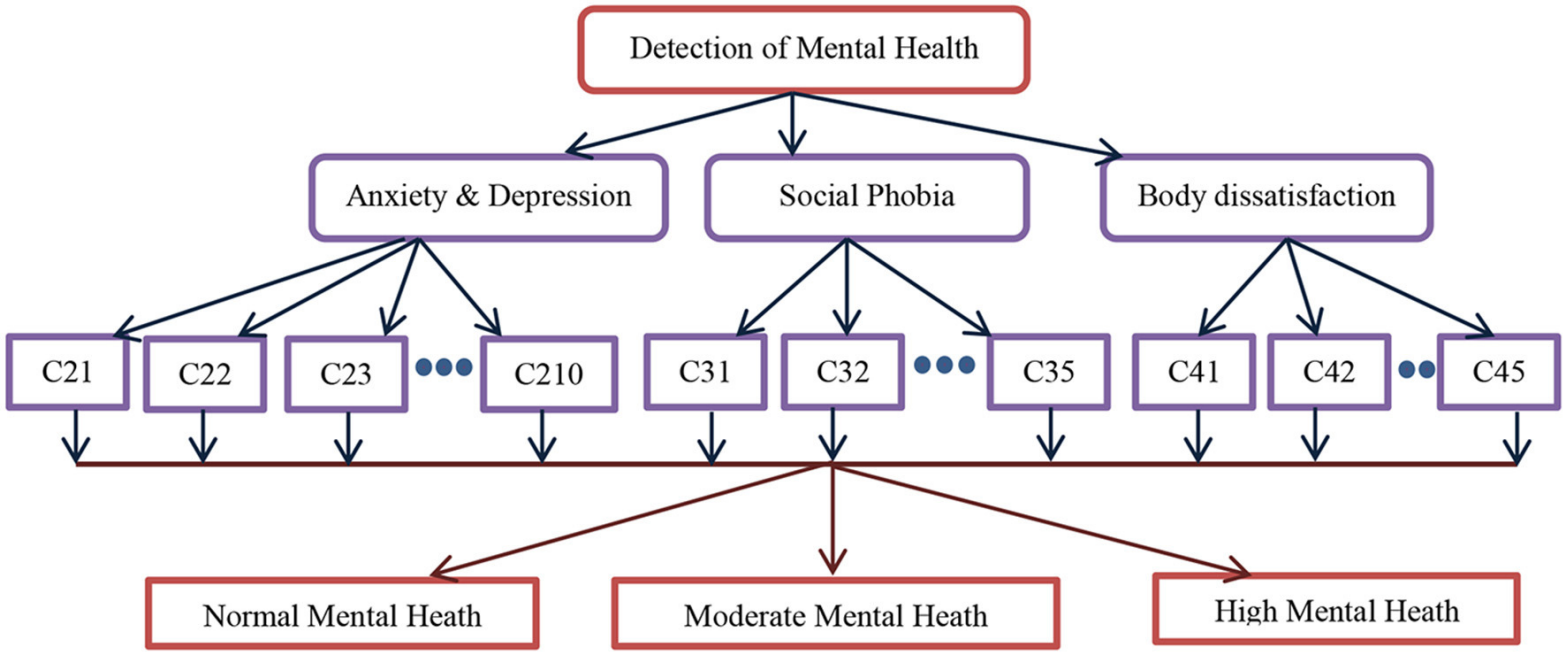
The computational model for the detection of mental health.

Figure 4 depicts the computational model to detect PCOS using physical parameters. As stated in the study design, in total 11 criteria from C11 to C111 are used as input in the computational model involving machine learning algorithms and multi-criteria decision systems such as fuzzy AHP followed by the fuzzy TOPSIS. The output of the computational model is discrete, viz, normal, moderate, and high PCOS problems. Similarly, Figure 5 shows the detection of mental health disorders using anxiety and depression, social phobia, and body dissatisfaction aspects. As depicted in Table 1 of the study design, C21–C45 are the criteria variables given as input to the computational models. In the same lines, the output is normal, moderate, and high mental health issues. The outcome of both physical aspects and mental health disorder are combined using fuzzy rules to provide final inference as abnormal having both PCOS and mental health, abnormal having only PCOS, Abnormal having only mental health issues, or Normal.

### Fuzzy AHP

**Figure 6:**
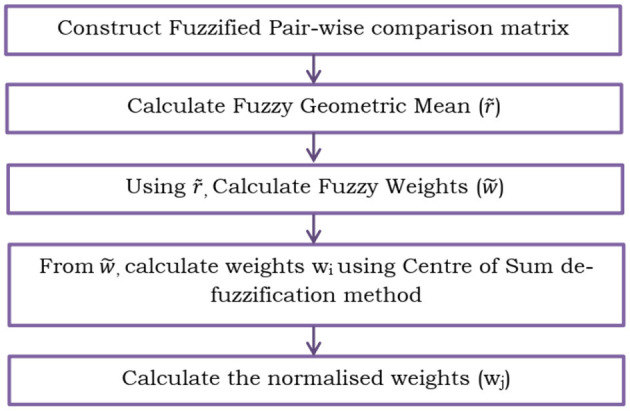
Fuzzy AHP.

In the proposed PCOS detection system, in order to quantify the weights in Fuzzy TOPSIS, Fuzzy AHP (30, 31) is used, which is depicted in Figure 6. The systematic steps are as follows:

**Step 1**: A criteria pair-wise comparison matrix M_ij_ is constructed. In total, 31 criteria are considered for the detection of PCOS and its associated mental health. M_ij_ is constructed by comparing the criteria in pairs to decide which among each pair is preferred or has a greater amount of quantitative property, or whether or not two of the criteria are identical. For this research work, the M_ij_ is constructed with the linguistic variables and its associated triangular fuzzy numbers as shown in Table 2.
**Step 2:** Construct the fuzzified geometric mean value. It is calculated as follows,
  (1) $$\begin{array}{r} M_{ij}={\left(x_{1}\times x_{2}\times x_{3}\times \ldots x_{n}\right)}^{\frac{1}{n}}\times {\left(y_{1}\times y_{2}\times y_{3}\times \ldots y_{n}\right)}^{\frac{1}{n}}\\ \;\times {\left(z_{1}\times z_{2}\times z_{3}\times \ldots z_{n}\right)}^{\frac{1}{n}} \end{array}$$
  where *x*_1_…*x*_*n*_ are the first elements in the fuzzy triangular numbers, *y*_1_…*y*_*n*_ are the second elements in the fuzzy triangular numbers, similarly, *z*_1_…*z*_*n*_are the third elements in the fuzzy triangular numbers, and *n* is the number of criteria, i.e., *n* = 31.
**Step 3:** Calculate the fuzzy weights ($\tilde{w}$) using the formula,
  (2) $$\begin{array}{r} w_{i}=r_{i}\times {\left(r_{1}+r_{2}+r_{3}\ldots r_{n}\right)}^{-1} \end{array}$$
  where *r*_1_ is the addition of the first column of the fuzzified geometric mean value, similarly, *r*_2_ and *r*_*n*_ is the addition of second and nth columns of the fuzzified geometric mean value, respectively.
**Step 4**: Any defuzzification method can be used to calculate the defuzzified weights (*w*_*i*_). For the proposed work, the Center of Area (COA) method is used.
  (3) $$\begin{array}{r} COA=\frac{\left(a+b+c\right)}{3} \end{array}$$
  Where a, b, and c are the 3 sides of the triangle formed by the triangular membership function.
**Step 5**: From the weights (*w*_*i*_), calculate normalized weights (*w*_*j*_),
  (4) $$\begin{array}{r} w_{j}=\frac{w_{i}}{\sum_{i=1}^{n}c_{i}} \end{array}$$
  These normalized weights are used to assign weights for the TOPSIS method.

### Fuzzy TOPSIS

**Figure 7:**
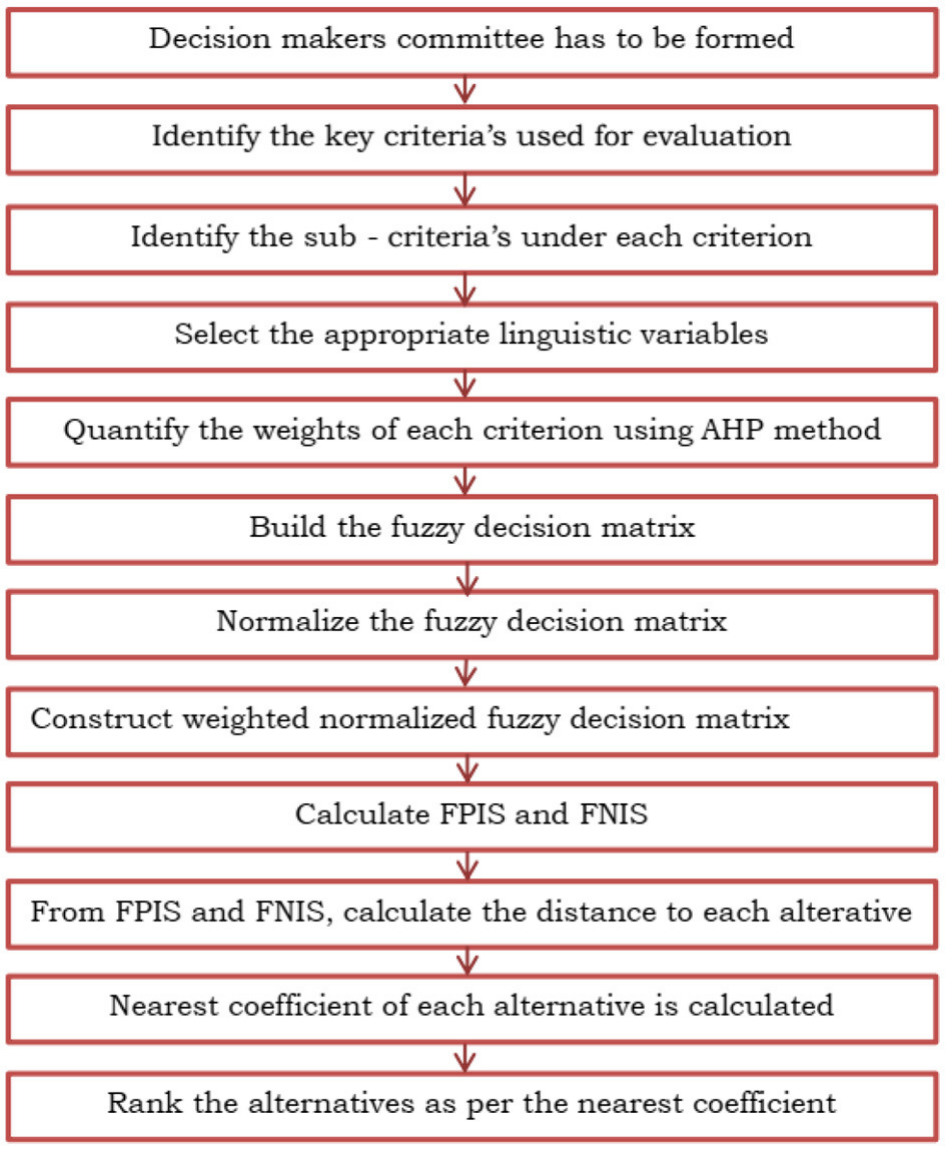
The flow of the Fuzzy TOPSIS method.

The Fuzzy TOPSIS method (32) is used which is described as follows:

**Step 1:** Initially the committee of decision-makers is formed. For this research work, the decision-makers are expert doctors in the gynecology department and the psychology department. The fuzzy rating of each decision-maker *D*_*k*_ *where*(*k* = 1, 2, 3) can be expressed as a triangular fuzzy number ${\tilde{p}}_{k}$ where (*k* = 1, 2, 3) with membership function $\mu \times {\tilde{p}}_{k}\left(x\;\right)$.
**Step 2:** Identify the key criteria and sub-criteria for evaluation.
**Step 3:** Select the suitable linguistic variables for evaluating the parameters such as criteria and alternatives. Tables 3, 4 show the linguistic variables and their associated fuzzy numbers for each criterion and alternatives.
**Step 4:** Quantify the weights for each criterion based on the values obtained from the AHP method. Using triangular fuzzy numbers, the decision-makers ratings are described as follows,
  ${\tilde{p}}_{k}=\left(m_{k},n_{k},o_{k}\right)$, where *k* = (1, 2, 3), then the total fuzzy rating can be determined as $\tilde{p}\;=\left(m,\;n,\;o\right)$ where,
  (5) $$\begin{array}{r} m={\text{min}}_{k}\left\{m_{k}\right\},\;n=\frac{1}{k}\overset{k}{\underset{k=1}{\sum }}n_{k},o={\text{max}}_{k}\left\{o_{k}\right\} \end{array}$$
  If the fuzzy ratings and the weights of k^th^ decision-makers are
  (6) $$\begin{array}{r} {ỹ}_{ijk}=\left(m_{ijk},n_{ijk},o_{ijk}\right)\text{ and }{\tilde{w}}_{ijk}=\left(w_{jk1},w_{jk2},w_{jk3}\right) \end{array}$$
  where i = 1, 2, 3 … m and j = 1, 2, 3,……n., then the total fuzzy rating (ỹ_*ij*_) of alternative will be
  $$\begin{array}{l} {ỹ}_{ij}=\left(m_{ij},n_{ij},o_{ij}\right) \end{array}$$
  where
  (7) $$\begin{array}{r} m_{ij}={\text{min}}_{k}\left\{m_{ijk}\right\},n_{ij}=\frac{1}{k}\overset{k}{\underset{k=1}{\sum }}n_{ijk},o_{ij}={\text{max}}_{k}\left\{o_{ijk}\right\} \end{array}$$
  ${\tilde{w}}_{ij}$ is the aggregated fuzzy weights of each criterion and is given by ${\tilde{w}}_{j}=$ (*w*_*j*1_, *w*_*j*2_, *w*_*j*3_) where
  (8) $$\begin{array}{r} w_{j1}={\text{min}}_{k}\left\{w_{jk1}\right\},w_{j2}=\frac{1}{k}\overset{k}{\underset{k=1}{\sum }}w_{jk2},w_{j3}={\text{max}}_{k}\left\{w_{jk3}\right\} \end{array}$$
**Step 5**: The fuzzy decision matrix is constructed as
  $$\begin{array}{l} \tilde{D}=\begin{array}{ccc} {ỹ}_{11} & {ỹ}_{12} & {ỹ}_{1n}\\ {ỹ}_{21} & {ỹ}_{22} & {ỹ}_{2n}\\ {ỹ}_{m1} & {ỹ}_{m2} & {ỹ}_{mn}\\ \; \end{array} \end{array}$$
  $\tilde{w}=\left[{\tilde{w}}_{1},{\tilde{w}}_{2},\ldots {\tilde{w}}_{n}\right]$. Here, ỹ_*ij*_ and ${\tilde{w}}_{j}$ can be approximated by the positive triangular fuzzy numbers.
**Step 6:** Using linear scale transformation, the values of the criteria are transformed from a criteria scale into a comparable scale as
  (9) $$\begin{array}{r} Ã={\left[{ã}_{ij}\right]}_{m\times n}\text{ where}\\ \;{ã}_{ij}=\left(\frac{m_{ij}}{c_{j}^{*}},\frac{n_{ij}}{c_{j}^{*}},\frac{o_{ij}}{c_{j}^{*}}\right),c_{j}^{*}={\text{max}}_{i}\left(c_{ij}\right) \end{array}$$
**Step 7:** The normalized weighted decision matrix is calculated. The weighted normalized decision matrix (ṽ) is calculated as
  (10) $$\begin{array}{r} \;ṽ={\left[{ã}_{ij}\right]}_{m\times n}\\ \text{where},{ã}_{ij}={ã}_{ij}\times {\tilde{w}}_{j} \end{array}$$
**Step 8:** Then the fuzzy positive ideal solution (FPIS,B^*^) and fuzzy negative ideal solution (FNIS, *B*^−^) are calculated as
  (11) $$\begin{array}{r} \;B^{*}=\left({ã}_{1}^{*},{ã}_{2}^{*},\ldots {ã}_{n}^{*}\right)\\ \;B^{-}=\left({ã}_{1}^{-},{ã}_{2}^{-},\ldots {ã}_{n}^{-}\right)\\ \text{where }{ã}_{j}^{*}={\text{max}}_{i}\left\{v_{ij3}\;\right\}\\ \text{where }{ã}_{j}^{-}={\text{min}}_{i}\left\{v_{ij1}\;\right\} \end{array}$$
**Step 9:** The distance from the alternative to the FPIS and FNIS as
  (12) $$\begin{array}{r} d_{i}^{*}=\overset{n}{\underset{j=1}{\sum }}\left({ã}_{ij},{ã}_{j}^{*}\right)\times d_{v}\text{ where }i=1,2,\ldots m \end{array}$$
  (13) $$\begin{array}{r} d_{i}^{-}=\overset{n}{\underset{j=1}{\sum }}\left({ã}_{ij},{ã}_{j}^{-}\right)\times d_{v}\text{ where }i=1,2,m \end{array}$$
  where *d*_*v*_ is the distance between 2 fuzzy numbers.
**Step 10:** The closeness coefficient (*CC*_*i*_) is calculated as
  (14) $$\begin{array}{r} CC_{i}=\frac{d_{i}^{-}}{d_{i}^{*}+d_{i}^{-}} \end{array}$$
**Step 11:** The physical and mental health are obtained in the decreasing order of the closeness coefficient values. The entire flowchart of the TOPSIS method is shown in Figure 7.

**Table 2:** Linguistic variables and their fuzzy numbers.

**Sl. No**	**Linguistic variables**	**Fuzzy triangular numbers**
1.	Equal important	(1, 1, 1)−1
2.	Moderate important	(2, 3, 4)−3
3.	Strong important	(4, 5, 6)−5
4.	Very strong important	(6, 7, 8)−7
5.	Extreme important	(9, 9, 9)−9
6.	Intermediate	2–(1, 2, 3), 4–(3, 4, 5), 6–(5, 6, 7), 8–(7, 8, 9)

**Table 3:** Linguistic variables for the importance weight of each criterion.

**Sl. No**	**Linguistic variables**	**Triangular fuzzy numbers**
1.	Very Less Relevant (VLR)	(0, 0, 2)
2.	Less Relevant (LR)	(2, 3, 4)
3.	Relevant (R)	(4, 5, 6)
4.	High Relevant (HR)	(6, 7, 8)
5.	Very High Relevant (VHR)	(8, 9, 10)

**Table 4:** Linguistic variables for alternatives.

**Sl. No**	**Linguistic variables**	**Triangular fuzzy numbers**
1.	Very Low (VL)	(0, 0, 0.2)
2.	Low (L)	(0.20, 0.3, 0.4)
3.	Medium (M)	(0.4, 0.5, 0.6)
4.	High (H)	(0.6, 0.7, 0.8)
5.	Very High (VH)	(0.8, 0.9, 1)

## Results

The proposed methodology using Fuzzy AHP and Fuzzy TOPSIS is implemented in python (Python Software Foundation. Delaware, United States). For quantifying the weights in fuzzy TOPSIS, fuzzy AHP is used. The normalized weights from the fuzzy AHP are shown in Tables 5A–C for a test example. As seen from Table 5A, it is observed that the weights C11, C12, C13, C14, and C16 have higher values compared with C15, C17, C18, C19, C110, and C111. Higher values for the weights indicate the relatively higher importance of those criteria. From Table 5B, it can be seen that C21, C22, C23, C25, and C27 have higher importance since the weights have higher values. In the same lines, as observed from Table 5C, C41 and C42 have higher weights and hence, these criteria have more importance compared with other criteria. Hence it is validated that, C11, C12, C13, C14, C16, C21, C22, C23, C25, C27, C41, and C42 are more important criteria in comparison with other criteria in making decision as given by fuzzy AHP. In the same way, the clinicians were also asked to provide the relative importance of the 31 criteria based on their clinical acumen. Their expert opinion also exactly matched with the outcome of the fuzzy AHP. Hence these weights are used for assigning the values in the fuzzy TOPSIS.

**Table 5A:** Weights for the criteria from C11 to C111.

**C11**	**C12**	**C13**	**C14**	**C15**	**C16**	**C17**	**C18**	**C19**	**C110**	**C111**
0.074	0.074	0.074	0.074	0.038	0.068	0.036	0.036	0.036	0.023	0.023

**Table 5B:** Weights for the criteria from C21 to C210.

**C21**	**C22**	**C23**	**C24**	**C25**	**C26**	**C27**	**C28**	**C29**	**C210**
0.045	0.045	0.045	0.023	0.029	0.04	0.021	0.012	0.012	0.012

**Table 5C:** Weights for the criteria from C31 to C35 and C41 to C45.

**C31**	**C32**	**C33**	**C34**	**C35**	**C41**	**C42**	**C43**	**C44**	**C45**
0.017	0.017	0.017	0.017	0.017	0.019	0.019	0.014	0.014	0.006

From equations 12 and 13, the distance between FPIS and FNIS for different criteria is computed and is given in Tables 6–8. The values obtained in these tables are used to compute the closeness coefficients. Table 9A provides the closeness coefficients between FPIS and FNIS for PCOS. A higher closeness coefficient value indicates a high probability of having PCOS. It can be seen that CC_1_ is higher compared with CC_3_ and CC_2._ Hence, it is observed that P1 (high PCOS) > P3 (moderate PCOS) > P2 (normal PCOS), therefore it is inferred that the possible condition is high PCOS for this particular test example. Similarly, Table 9B, shows the closeness coefficients between FPIS and FNIS for mental health. It is observed that M1 (high mental health) > M3 (moderate mental health) > M2 (normal mental health), and therefore the inference is high mental health issues for the same test example.

**Table 6:** Distance between P^*^ and *P*_*i*_ (i = 1, 2, 3), *P*^−^ and *P*_*i*_ (i = 1, 2, 3) for the criteria from C11 to C110.

$\text{d}({\text{P}}_{\text{i}},{\text{P}}^{*}$)	**C11**	**C12**	**C13**	**C14**	**C15**	**C16**	**C17**	**C18**	**C19**	**C110**	**C111**
d(P, P^*^)	2.35	2.35	3.34	2.35	2.95	4.24	2.95	2.3	2.3	3.1	3.1
$\text{d}\left(P_{2},P^{*}\right)$	9.38	9.38	8.54	9.38	6.6	8.27	6.6	5.4	5.4	4.08	4.08
$\text{d}\left(P_{3},P^{*}\right)$	4.8	4.8	6.43	4.97	4.08	5.46	4.08	3.38	3.38	2.42	2.42
d(P_i_, *P*^−^)	C11	C12	C13	C14	C15	C16	C17	C18	C19	C110	C111
d(*P*_1_, *P*)	8.3	8.3	7.72	8.3	5.94	7.17	5.94	4.91	4.91	2.72	2.72
$\text{d}\left(P_{2},P^{-}\right)$	1.15	1.15	2.37	1.15	2.02	2.5	2.02	1.57	1.57	1.73	1.73
$\text{d}\left(P_{3},P^{-}\right)$	5.96	5.96	4.22	5.79	4.57	5.55	4.57	4.34	4.34	3.6	3.6

**Table 7:** Distance between M^*^ and *M*_*i*_ (i = 1, 2, 3), *M*^−^ and *M*_*i*_ (i = 1, 2, 3) for the criteria from C21 to C210.

**d(M_i_, M)**	**C21**	**C22**	**C23**	**C24**	**C25**	**C26**	**C27**	**C28**	**C29**	**C210**
$\text{d}\left(M_{1},M^{*}\right)$	2.35	3.33	3.33	2.09	3.34	3.33	2.84	2.09	3.24	3.07
d(*M*_2_, *M*)	9.38	9.38	9.38	7.5	8.54	9.38	6.84	7.5	7.5	7.5
$\text{d}\left(M_{3},M^{*}\right)$	4.8	5.3	5.3	3.95	4.97	5.3	4.08	3.95	4.59	4.46
d(M_i_, *M*^−^)	C21	C22	C23	C24	C25	C26	C27	C28	C29	C210
$\text{d}\left(M_{1},M^{-}\right)$	8.3	7.73	7.73	6.5	7.72	7.73	6.07	6.5	5.78	5.96
$\text{d}\left(M_{2},M^{-}\right)$	1.15	1.15	1.15	0.92	2.37	1.15	1.89	0.92	0.92	0.92
$\text{d}\left(M_{3},M^{-}\right)$	5.96	5.7	5.7	4.7	5.79	5.7	4.57	4.7	4.33	4.45

**Table 8:** Distance between M^*^ and *M*_*i*_ (i = 1, 2, 3), *M*^−^ and *M*_*i*_ (i = 1, 2, 3) for the criteria from C31 to C35 and C41. C45.

$\text{d}({\text{M}}_{\text{i}},{\text{M}}^{*}$)	**C31**	**C32**	**C33**	**C34**	**C35**	**C41**	**C42**	**C43**	**C44**	**C45**
$\text{d}\left(M_{1},M^{*}\right)$	2.84	2.84	2.84	2.84	2.84	2.84	3.49	3.22	2.6	2.82
$\text{d}\left(M_{2},M^{*}\right)$	6.84	6.84	6.84	6.84	6.84	8.54	8.54	6.3	4.75	1.89
$\text{d}\left(M_{3},M^{*}\right)$	3.95	3.95	3.95	3.95	3.95	4.8	4.8	3.38	2.71	2.76
d(M_i_, *M*^−^)	C31	C32	C33	C34	C35	C41	C42	C43	C44	C45
$\text{d}\left(M_{1},M^{-}\right)$	6.07	6.07	6.07	6.07	6.07	7.55	7.55	5.88	4.31	1.23
$\text{d}\left(M_{2},M^{-}\right)$	1.89	1.89	1.89	1.89	1.89	2.37	2.37	2.52	1.88	2.4
$\text{d}\left(M_{3},M^{-}\right)$	4.7	4.7	4.7	4.7	4.7	5.96	5.96	5.71	4.19	1.26

**Table 9A:** Closeness coefficient for PCOS for the alternatives *P1, P2*, and *P3*.

**CC_**i**_ for PCOS**	**Closeness coefficient**	**Alternative**
*CC1*	*0.68*	*P1*
*CC2*	*0.53*	*P3*
*CC3*	*0.2*	*P2*

**Table 9B:** Closeness coefficient for mental health for the alternatives *M1, M2*, and *M3*.

**CC_**i**_ for mental health**	**Closeness coefficient**	**Alternative**
*CC1*	*0.68*	*M1*
*CC2*	*0.54*	*M3*
*CC3*	*0.19*	*M2*

Based on the individual (both physical and psychological) closeness coefficients, the fuzzy rules are framed in consultation with the clinicians. The rules are given in Table 10.

**Table 10:** Fuzzy inference rules.

**Sl. No**	**Fuzzy rule base for inference**
1.	If P1 & M1 then A1 (having high PCOS and high Mental health)
2.	If P1 & M2 then A2 (having high PCOS and normal Mental health)
3.	If P1 & M3 then A1 (having high PCOS and moderate Mental health)
4.	If P2 & M1 then A3 (having normal PCOS and high Mental health)
5.	If P2 & M2 then A4 (having normal PCOS and normal Mental health)
6.	If P2 & M3 then A3 (having normal PCOS and moderate Mental health)
7.	If P3 & M1 then A1 (having moderate PCOS and high Mental health)
8.	If P3 & M2 then A2 (having moderate PCOS and normal Mental health)
9.	If P3 & M3 then A1 (having moderate PCOS and moderate Mental health)

Similarly, machine learning algorithms, D-tree, KNN, and SVM algorithms are used to predict PCOS and its associated mental health issues independently and the final inference is drawn as per the rules provided in Table 9. In this process, the entire dataset is divided into a training set (80%) and testing set (20%).

The confusion matrix due to the classification using SVM and fuzzy TOPSIS is provided in Table 11 respectively. It can be noted that the Fuzzy TOPSIS method provides the highest performance compared with all other classifiers.

**Table 11:** Confusion matrix during support vector machines (SVM) classification (left) and fuzzy technique for order of preference by similarity to ideal solution (TOPSIS) (right).

Actual class	Predicted class A1 A2 A3 A4 A1[[ 37 0 0 0] A2 [ 0 24 0 0] A3 [ 1 0 38 4] A4 [ 0 3 2 58]]	Actual class	Predicted class A1 A2 A3 A4 A1[[ 37 0 0 0] A2 [ 0 24 0 0] A3 [ 0 0 41 2] A4 [ 0 0 1 62]]

The number of classes defines the size of the confusion matrix. In the current study, there are four classes in the data and hence, the confusion matrix is 4 × 4. The true positive (TP), false positive (FP), true negative (TN), and false negative (FN) values are evaluated as follows.

TP of current class = Principal diagonal values of current row/column.

FP of current class = (Sum of current row values–TP of current class).

FN of current class = (Sum of current column values–TP of current class).

TN of current class = Sum of the matrix–TP of current class–Sum of current row values–Sum of current column values.

Tables 12, 13 present the TP, FP, and FN for SVM and the fuzzy TOPSIS classifier, respectively.

**Table 12:** Components of confusion matrix for SVM.

**Class**	**A1**	**A2**	**A3**	**A4**
True positive	37	24	38	58
False positive	0	0	5	5
False negative	1	3	2	4

**Table 13:** Components of confusion matrix for fuzzy TOPSIS.

**Class**	**A1**	**A2**	**A3**	**A4**
True positive	37	24	41	62
False positive	0	0	2	1
False negative	0	0	1	2

It can be seen from Table 12 that there are 10 FP and 10 FN during SVM classification, whereas during the fuzzy TOPSIS method, as indicated in Table 13, there are only three FP and three FN. It is noted that fuzzy TOPSIS provides less FN as compared with other classifiers. The presence of FN is crucial. It means that the woman actually with PCOS are diagnosed as not having PCOS by the automated system. It also indicates that the particular woman actually with PCOS goes undiagnosed, which is a fatal error. It can be inferred that Fuzzy TOPSIS provides less FN and hence, it is superior compared with all other classifiers. On the other hand, fuzzy TOPSIS has provided less FP compared with other classifiers. False-positive means a woman does not actually have PCOS but the automated system diagnoses her with PCOS. This causes a lot of undue panic and anxiety to the patients. Hence the fuzzy TOPSIS is superior in terms of less FP as well.

**Figure 8:**
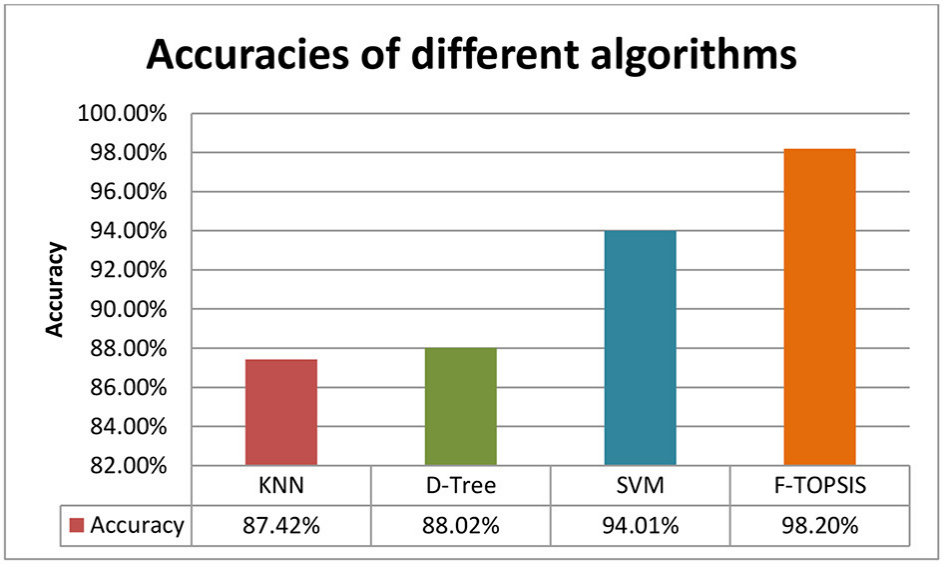
Model evaluation: accuracies of different classifier.

The accuracy of classification using KNN, D-tree, SVM, and fuzzy TOPSIS is shown in Figure 8. It can be noted that the SVM provides an accuracy of 94.01%, and D-tree and KNN provide 88.02 and 87.42%, respectively. It is observed that SVM outperforms the D-tree and KNN. The fuzzy TOPSIS provides an accuracy of 98.20%, outperforming all other classifiers.

It is also interesting to assess the mental health issues of a woman who was diagnosed with PCOS. Table 14 provides the proportion of women with PCOS in the testing data, as well as the proportion of women with mental health issues in the test data. It can be seen that the fuzzy TOPSIS provided that 52 women have a high possibility of PCOS and four women have a moderate possibility of PCOS. So in total, 56 women have PCOS.

**Table 14:** The proportion of women having PCOS and mental health issues in the testing data.

**#Testing data**	**PCOS**		**Mental health**	
	**P1 (High possibility)**	**P3 (Moderate)**	**M1 (High Possibility)**	**M3 (Moderate)**
167	52	4	83	1

From this data, the mental wellness indicator (I_M_) of PCOS patients is defined as,

$$\begin{array}{l} I_{\text{M}}=\frac{n\;\left\{\left(P1\;\vee \;P3\right)\;\wedge \;\left(A1\right)\right\}}{n\;\left\{P1\;\vee \;P3\right\}}\times 100 \end{array}$$

where ‘*n*’ represents the count meeting the condition.

Similarly, the physical wellness indicator (I_P_) of a patient suffering from mental illness is defined as,

$$\begin{array}{l} I_{\text{P}}=\frac{n\;\left\{\left(M1\;\vee \;M3\right)\;\wedge \;\left(A1\right)\right\}}{n\;\left\{M1\;\vee \;M3\right\}}\times 100 \end{array}$$

**Figure 9:**
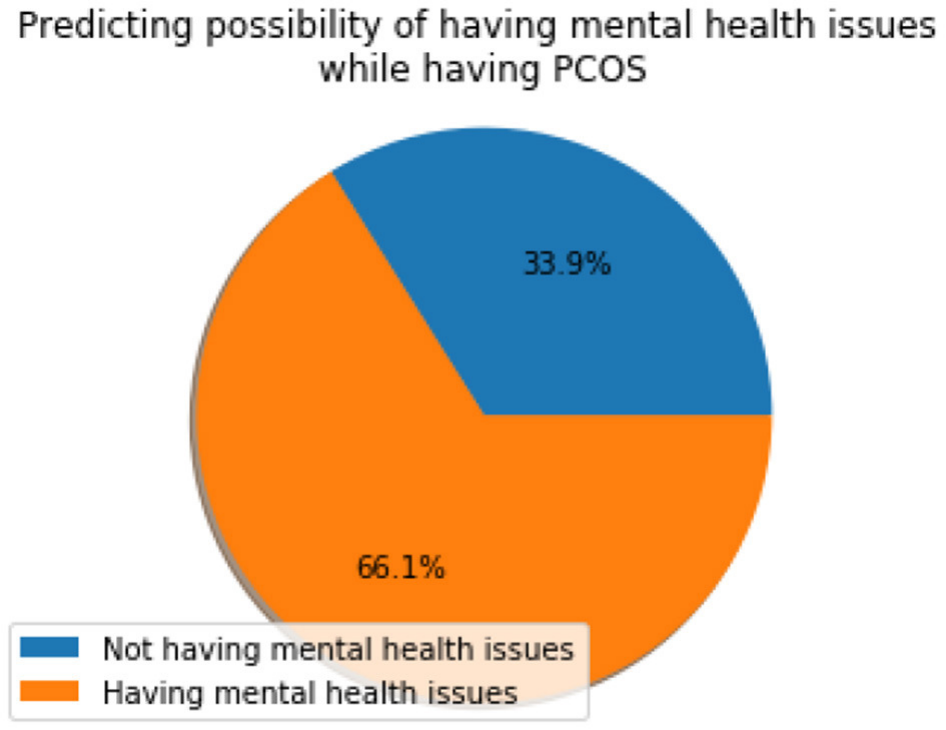
Mental wellness indicator (I_M_).

Figure 9 shows the mental wellness indicator (I_M_) which resulted in a value of 66.07%, which means the majority of women suffering from PCOS also suffer from mental health issues. This shows that there is a strong association between PCOS and the mental well-being of a woman which needs to be taken care of during the clinical investigation and diagnosis.

**Figure 10:**
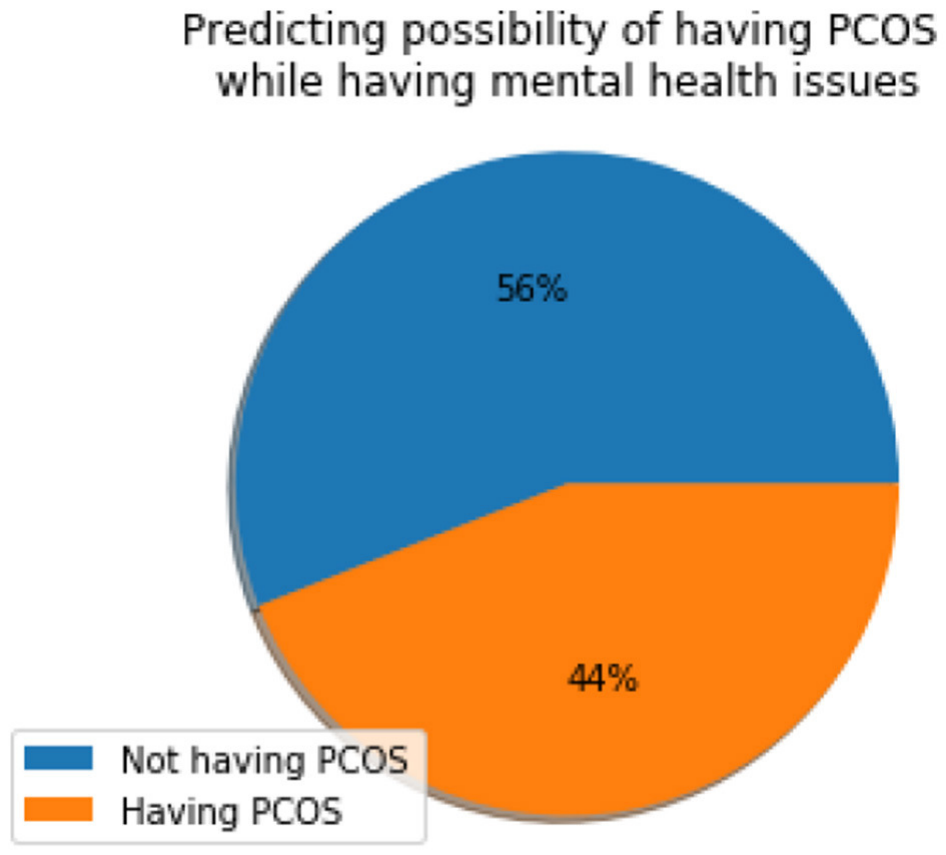
Physical wellness Indicator (I_P_).

Figure 10 depicts the physical wellness indicator (I_P_) which has a value of 44%, suggesting that a considerable proportion of women suffering from mental health also have PCOS. This information can be effectively used during clinical evaluation and appropriate diagnosis can be established.

## Discussion

The present study investigates the early prediction of PCOS and its associated mental health issues more objectively by incorporating inherent fuzziness in the decision process. The fuzzy algorithm TOPSIS outperformed the conventional methods. Further, the mental wellness parameters, such as anxiety, depression, social phobia, and body image dissatisfaction, are integrated with clinical parameters to assess the women for the prognosis and diagnosis of PCOS and its associated socio-psychological well-being as 66.07% of women with PCOS have associated mental health issue as per the results of this study.

The computer diagnosis/automation of the early prediction of PCOS can bring a paradigm change in the conventional practices in the management of PCOS. The incorporation of the computer algorithm in the diagnosis can speed up the process and it can help the doctors in delivering the diagnosis quickly and easily. The developed computer diagnosis methodology is faster and more efficient; more women can be screened for PCOS in a short periodof time. Hence this methodology can offer a low-cost and mass screening protocol to detect PCOS. This in turn can help women of low socio-economic conditions by providing a quick, efficient, and low-budget solution.

The regular screening of women for PCOS involves personal interaction, clinical examination, ultra-sound scans, etc. The developed methodology in this study involves a pre-designed questionnaire presented by the computer to the women, which comprises both physical and psychological health questions where the privacy of the women is taken care of. Women can feel free while answering the questions with the computer in their own comfort zone without invading their privacy.

It is interesting to compare our results with previously published research as shown in Table 15. Denny et al. (24), in his study, used multiple parameters such as ultrasound result (size of follicles, number of follicles), blood investigations (TSH, AMH, vitamin D3, etc.), and clinical features (cycle length and regularity), and obtained an accuracy of 89.02% using the random forest classifier. Since hormonal tests are involved in the data collection, the process becomes expensive in terms of cost. Further, Vikas et al. (25), in their study, considered parameters such as lifestyle and food intake habits, and psychological parameters like anxiety and depression, and obtained an accuracy of 97.65% using the Naïve Bayes classifier, however, the questionnaire involved binary (yes and no) responses. Using ultrasound scanned images, Anuradha and Priyanka (26) and Deshpande and Wakankar (27) investigated PCOS and obtained an accuracy of 98% using Artificial Neural Networks and 95% using SVM, respectively. Further, Meena et al. (33), using endometrial biopsies, obtained an accuracy of 83.70% using Artificial Neural Networks. However, in these studies, mental health parameters are not considered. Further, Satish et al. (34), using clinical and biochemical parameters such as BMI, pulse rate, hemoglobin, and hormonal tests (which include FSH, Prolactin, Progesterone), obtained an accuracy of 87.72% using the Naïve Bayes classifier, yet even in this study, mental health parameters are not considered. All these above studies involve complex and expensive parameters such as hormonal investigations, blood investigations, and ultrasound scanned images which are difficult to acquire, take a longer time, and are expensive, and hence, women with low socioeconomic status cannot afford them.

**Table 15:** Comparison of results with the previously published research.

**References**	**Dataset used**	**No of attributes**	**Parameters considered**	**Algorithms used**	**Performance**
Denny et al. (24)	541 women	23	Clinical and metabolic parameters such as no. of follicles, size of follicles, TSH, AMH, Vit D3, cycle length & regularity etc.	6 classification algorithms: CART, SVM, KNN, Logistic regression, Naïve Bayes, Random Forest	Accuracy of 89.02% by Random Forest classifier
Vikas et al. (25)	119 women	18	Life style and food intake habits such as regularity of cycle, anxiety & depression, mental stress	ANN, Naïve Bayes, Decision Tree	Accuracy of 97.65% by Naïve Bayes, 96.27% by ANN, 96.24% by Decision Tree
Anuradha and Priyanka (26)	84 women	13	Acne, irregular periods, sonography, LH & weight	ANN, KNN, & Linear regression	Accuracy of 94% by ANN
Deshpande and Wakankar (27)	20 women	5	clinical (BMI and cycle length), biochemical (FSH and LH levels) and imaging (calculating number of follicles present in the ovary).	SVM	Accuracy of 95% by SVM
Meena et al. (33)	303 women	26	Endometrial biopsies	Decision Tree, Naïve Bayes, SVM, ANN	Accuracy of 76.45% by SVM, 83.70% by ANN, 75.25% by D-Tree, 82.75% by Naïve Bayes
Satish et al. (34)	541 women	41	Clinical & biochemical parameters such as BMI, pulse rate, hemoglobin, hormonal tests include FSH, Prolactin, Progesterone	KNN, SVM, RF, GNB, ANN	Accuracy of 75.45% by KNN, 82.27% by SVM, 85% by RF, 87.72% by Naïve Bayes, 50% by ANN
Proposed work	629 women	33	Physical parameters (length and duration of the cycle etc.) and psychological parameters (anxiety, depression, body dissatisfaction and social phobia)	Fuzzy TOPSIS,SVM, KNN, Decision Tree	Accuracy of 98.20% by Fuzzy TOPSIS, 94.01% by SVM, 88.02% by D-Tree, 87.42% by KNN

Given the limitations of the above studies, this research involves simple regular parameters (physical and mental health), yet an improved performance of 94.01 and 98.20% with SVM and TOPSIS, respectively, are obtained. The present study provides higher performance than the existing standard and widely used methods. It also provides a new paradigm of PCOS diagnosis using computational intelligence by using the fuzzy TOPSIS method. In addition, the method also provides improved performance when compared with conventional methods. This methodology can also be used for the mass screening of the population by developing countries with large populations and constrained resources. Women can undergo this methodology in their own comfort zone without the intervention of a physician guarding their privacy.

The nature of data comprising of physical and mental health parameters is inherently imprecise, vague, and perceptually subjective. The traditional data analysis methods such as SVM, KNN, and the D-tree can model the crisp data accurately. When the data itself is imprecise, the crisp models cannot address the intricate fuzzy interrelations in the data. Hence the fuzzy set-based models can address the inherent subjectivity in the data and can provide improved performance. The fuzzy TOPSIS can be used to find out the most suitable alternative concerning different selection criteria. Also, the fuzzy distances used in fuzzy TOPSIS provide improved closeness coefficient values compared with other fuzzy measures. Therefore, the selected fuzzy TOPSIS has a higher potential in representing the physical phenomena of the scenario.

In addition to the above contributions, this research work also demonstrates the importance of the mental well-being of a woman during PCOS. The result indicates that the majority of women suffering from PCOS also undergo mental health issues. The outcome of this study recommends having mental health evaluations during the diagnosis of PCOS.

## Conclusion

The strength of the proposed automated system involves the inclusion of PCOS and mental health questionnaires which have been validated by expert gynecologists and psychiatrists. Considering anxiety, depression, social phobia, and body image dissatisfaction analysis along with physical aspects adds new knowledge to the literature. In this study, the multi-criteria decision analysis system is developed for the early prediction of PCOS problems and mental health before the progression of the disease which can reduce morbidity. The proposed fuzzy TOPSIS-based automated system provides a high performance of 98.20% in real-time scenarios. The inherent uncertainty and imprecision involved in the data and decision-making process are well-modeled by Fuzzy TOPSIS in comparison with other methods which is evident with the high performance obtained. In addition to methodological contributions and improved performance, the study also indicates that the majority of women suffering from PCOS also have associated mental health issues. The present study proved that 66.07% of women with PCOS have associated mental health issues. This strong association implies that there is a strong need for improved protocols and efficient stress and mental health balancing guidelines in the management of PCOS.

The developed system in this study can help physicians in their clinical practice by acting as an adjunct tool. The proposed method is cost-effective and hence, women with low socio-economic conditions can also avail the screening. As the screening is automated and does not involve the intervention of clinicians, women can use the tool in their own comfort level with privacy. The use of the automated algorithm integrated with minimal intervention from physicians can speed up the screening process, making the screening available for more women in a given time and can bring a change in the conventional practices.

## Data Availability Statement

The raw data supporting the conclusions of this article will be made available by the authors, without undue reservation.

## Author Contributions

All authors listed have made a substantial, direct and intellectual contribution to the work and approved it for publication.

## Conflict of Interest

The authors declare that the research was conducted in the absence of any commercial or financial relationships that could be construed as a potential conflict of interest.

## Publisher's Note

All claims expressed in this article are solely those of the authors and do not necessarily represent those of their affiliated organizations, or those of the publisher, the editors and the reviewers. Any product that may be evaluated in this article, or claim that may be made by its manufacturer, is not guaranteed or endorsed by the publisher.
